# Treatment of Sick Children Seeking Care in the Private Health Sector in Uganda: A Cluster Randomized Trial

**DOI:** 10.4269/ajtmh.19-0367

**Published:** 2020-01-20

**Authors:** Anthony K. Mbonye, Esther Buregyeya, Elizeus Rutebemberwa, Sham Lal, Siân E. Clarke, Kristian S. Hansen, Pascal Magnussen, Philip LaRussa

**Affiliations:** 1 School of Public Health, College of Health Sciences, Makerere University, Kampala, Uganda;; 2 Department of Disease Control and Environmental Health, School of Public Health, Makerere University, Kampala, Uganda;; 3 Department of Health Policy, Planning and Management, School of Public Health, Makerere University, Kampala, Uganda;; 4 Department of Disease Control, London School of Hygiene and Tropical Medicine, London, United Kingdom;; 5 Department of Public Health and Centre for Health Economics and Policy, University of Copenhagen, Copenhagen, Denmark;; 6 Institute for Immunology and Microbiology, Centre for Medical Parasitology, University of Copenhagen, Copenhagen, Denmark;; 7 Department of Pediatrics, College of Physicians and Surgeons, Columbia University, New York, New York

## Abstract

The main objective of this study was to assess whether training of private health providers and community sensitization on the importance of effective prompt care seeking and the need for referral could improve treatment of sick children in the private health sector in Uganda. Private providers were trained to diagnose and treat sick children according to the integrated community case management (iCCM) guidelines. In the control arm, routine services were offered. The outcomes were seeking care within 24 hours of onset of symptoms and appropriate case management for malaria, pneumonia, and diarrhea among children aged < 5 years. A total of 10,809 sick children (5,955 in the intervention arm and 4,854 in the control arm) presented for diagnosis and treatment. The percentage seeking care within 24 hours of onset of symptoms was 45.4% (95% CI 36.0–48.8) in the intervention arm versus 43.9% (95% CI 38.1–49.8) in the control arm (*P* = 0.04). Adherence to malaria rapid diagnostic test (mRDT) results was high, with 1,459 (94.3%) in the intervention arm versus 1,402 (83.0%) in the control arm (*P* = 0.04). Appropriate treatment of mRDT-positive children with artemisinin-based combination therapy was seen in 93.1% (95% CI 88.5–97.7) in the intervention arm versus 85.1% (95% CI 78.6–91.7) in the control arm (*P* = 0.03). Adherence to iCCM guidelines was very high: 89.1% of children with diarrhea in the intervention arm and 80.4% in the control arm were given oral rehydration salts and zinc (*P* = 0.01). Of the children with a respiratory rate > 40 breaths/minute, 1,596 (85.1%) in the intervention arm versus 104 (54.5%) in the control arm were given amoxicillin (*P* = 0.01). In conclusion, the intervention improved treatment of malaria, pneumonia, and diarrhea because of provider adherence to treatment guidelines. The policy implications of these findings are to initiate a dialogue at district and national levels on how to scale up the intervention in the private sector. NCT02450630 registered with ClinicalTrials.gov: May 9, 2015.

## INTRODUCTION

Although there has been a decline in under-five mortality in Uganda during the last 5 years from 90 to 64 per 1,000 live births,^1^ there is need for further reduction in early childhood infections because the disease burden remains high. In a recent survey, 30% of children aged less than 5 years reported fever and 26% reported upper respiratory tract infections.^2^ Management of sick children at private health facilities has been shown to be poor, with only 10.3% of febrile children treated at drug shops receiving appropriate treatment for malaria, 16% of children with both cough and fast breathing receiving amoxicillin, and 14% of children with diarrhea receiving oral rehydration salts (ORS), with none receiving zinc tablets. The study concluded that management of common childhood illnesses at private sector drug shops in rural Uganda was largely inappropriate.^3^

Yet, it has been shown that approximately 48% of people with illnesses in Uganda seek care from private clinics, drug shops, and pharmacies as the first resort.^3^ Given this scenario, we conceptualized that training providers in this sector might improve case management of the three major childhood illnesses (malaria, pneumonia, and diarrhea). Previous studies have only evaluated interventions in drug shops and have not assessed the impact of training other private providers to diagnose and treat malaria, pneumonia, and diarrhea beyond drug shops.^3–8^

To improve treatment of sick children in the private health sector, we designed a study with the main objective of training providers to adhere to integrated community case management (iCCM) treatment guidelines in private health facilities.^9^ We hypothesized that training private healthcare providers on appropriate treatment of common childhood illnesses and raising community awareness would lead to improved treatment and uptake of referral advice. The primary outcome was the proportion of sick children appropriately treated according to the treatment guidelines, whereas the secondary outcome was prompt care seeking within 24 hours of onset of symptoms.

## MATERIALS AND METHODS

### Trial design and study setting.

The study was conducted in Mukono district, central Uganda. The background characteristics of the study area have been published elsewhere.^10–13^ In brief, the total population of the district was 583,600, with 88% living in rural settlements. The district is mainly inhabited by the Baganda ethnic group practicing subsistence agriculture.^14^^,^^15^ Mukono district is endemic for malaria, pneumonia, and diarrhea. It has numerous registered drug shops and private clinics that have been shown to be able to diagnose malaria with malaria rapid diagnostic tests (mRDTs) and treat it appropriately with artemisinin-based combination therapy (ACT).^8^ The target population was sick children (< 5 years) who sought treatment at private outlets (private clinics and registered drug shops).

### Description of the intervention.

The intervention had three components in the intervention arm: 1) training private healthcare providers to diagnose, treat, and refer sick children according to the iCCM guidelines; 2) community sensitization by village health teams (VHTs) to initiate community discussions aimed at supporting prompt treatment seeking and referral; and 3) health system strengthening by organizing regular meetings between the public and private healthcare providers (convened by the district health team) to discuss health system issues focusing on treatment and the referral system. In the control arm, routine services were provided, with providers trained on data capture with patient registers, treatment, and referral forms.

The majority of providers—87.7% at drug shops and 56.0% at private clinics—were female. All providers at drug shops and 93.2% at private clinics had attained secondary education. A large proportion of health providers at drug shops were enrolled as nurses/midwives (lower cadre who get a certificate after secondary school), whereas at private clinics, providers were registered nurses/midwives and clinical officers (higher cadre with a diploma). Overall, 55.6% health providers had been trained on malaria, 12.5% on pneumonia, and 35.3% on diarrhea management (data not shown).

### Training of healthcare providers.

There was no training given to providers in the control arm. In the intervention arm, however, training of providers took place in May 2015 for 4 days and was based on a trainer’s manual and an accompanying set of pictorial job aids. Training encouraged interactive discussions and reflective practice, supported by role-plays to practice skills necessary to obtain clinical history, explain outcome of tests, treatment given, and referral advice. The training included the following: educating 1) how to recognize signs and symptoms to distinguish uncomplicated malaria (fever for less than 7 days, with no danger sign) from severe malaria and provide an age-dependent dose of ACT–artemether–lumefantrine (Coartem^®^, Cipla Ltd., India/Uganda) to children with uncomplicated malaria, as well as administer rectal artesunate for pre-referral treatment of severe malaria; and 2) how to recognize cough and rapid breathing and prescribe amoxicillin, signs and symptoms of diarrhea, and administer ORS and zinc tablets to children. For cough, the recommendation was that fast breathing was a sign of pneumonia, and a child with fast breathing must be given an antibiotic. A child aged 2 months to 5 years who had a cough and fast breathing for less than 21 days, with no danger signs, was to be treated with amoxicillin. Fast breathing was assessed using the following respiratory rate cutoffs per age: 0–59 days, > 60 breaths per minute; 2–11 months, > 50 breaths per minute; and 12–59 months, > 40 breaths per minute. The dosage of amoxicillin for each age group and duration of treatment was given in the job aid. Adherence to these recommendations were verified through examination of data on history at presentation, signs and symptoms recorded, treatment given, and cases of referral.

For diarrhea, the recommendation was as follows: a child with loose stools for less than 14 days with no blood in stool and no other danger signs was to be given ORS solution and a zinc supplement. It was explained that a child with diarrhea could quickly become dehydrated and die because the body loses water and salts with diarrhea, and these must be replaced. Giving water, breast milk, and other fluids to children with diarrhea helps prevent dehydration. This may include light locally available porridges and coconut water.^16^ A pre- and posttest were administered, and all trainees passed the posttest.

Private providers were given low literacy visual job aids, explaining symptoms, diagnosis, correct treatment of malaria, pneumonia and diarrhea, and danger signs which could be used to explain the pictorial instructions to caregivers, as well as give instructions on dosing and how to prescribe drugs to patients. They were also given treatment forms, referral forms, timers, basic drugs, and supplies.

The age and gender of children, onset of symptoms and promptness of treatment-seeking, diagnosis—including mRDT result, tablets supplied, and the outcome (treatment and/or referral) were routinely recorded by private outlets for children seeking treatment for fever, cough, or diarrhea.

Healthcare providers were trained to give pre-referral treatment using a rectal artesunate suppository to children with danger signs consistent with suspected severe malaria^16^ and to refer these cases immediately to the nearest formal health unit for further treatment. Training also included referral of any mRDT-negative sick children with danger signs and/or a temperature > 38.5°C to the nearest health unit as an emergency case. This additional temperature criterion was based on the WHO definition of hyperthermia and is intended to help ensure that those with severe bacterial infections are referred^16^—and this was added to the iCCM training for the private providers.

An emergency referral form, including details of symptoms and the use of pre-referral treatment was provided to the care provider. They were trained to advise on mRDT-negative children with fever, cough, diarrhea (axillary temperature ≥ 37.5°C), or other symptoms (non-malaria), to seek treatment at the nearest health facility for further evaluation by a fully qualified health worker. A standard referral form was used for this purpose. For patients who were mRDT negative with no serious symptoms, private healthcare providers were trained to advise parents on tepid sponging, but to return for further assessment at the private healthcare facility or a public health facility should the symptoms persist or worsen.

Appropriate case management for fever cough and diarrhea was defined as treatment given according to the iCCM guideless as follows^17^: a child with cough less than 21 days with fast breathing was treated with an antibiotic (amoxicillin) as follows—a child aged 2–11 months was given two tablets twice a day for 5 days; a child aged 1–5 years was given three tablets twice a day for 5 days. A child with diarrhea for less than 14 days with no blood in stools was treated with ORS to replace the fluids and prevent dehydration and was also given zinc to lessen the amount of fluid lost and shorten the number of days of diarrhea, as follows: a child aged 2–6 months was given half a tablet of zinc once daily for 10 days, a child aged 7 months–5 years was given one tablet of zinc once daily for 10 days. A child with fever for less than 7 days with an mRDT-positive test was treated with an ACT (artemether–lumefantrine) as follows: a child aged 4 months–2 years was given one tablet of artemether–lumefantrine (Coartem) twice a day for 3 days; a child aged 3–5 years was given three tablets twice a day for 3 days.

### Supervision.

For the first 2 months of implementation, private providers in both arms received weekly supervision visits by members of the research team and a field supervisor to ensure adoption of the new procedures and promote accurate and complete record keeping. Pictorial job aids and detailed standard operating procedures (SOPs) were provided for quality assurance to ensure comparability. Providers were given the supervisors’ telephone number to contact them whenever they encountered any difficulties.

Case management skills of private providers were assessed during training and 2 months into implementing the intervention. Standard operating procedures and job aids were provided to support the skills of providers. After 2 months of the intervention, supportive supervision was scaled down to private facilities who occasionally experienced problems and asked for help. This was done to minimize the risk that the evaluation might influence provider behavior (Hawthorne effect). Nonetheless, a field supervisor was employed who regularly visited private providers and was ready to offer support when needed. In addition, providers’ skills were evaluated through a validation of patient registers, treatment, and referral forms.

### Community sensitization.

In the intervention arm, 3 months of community consultation and sensitization took place before implementing the intervention to increase awareness within the community about effective prompt care seeking for sick children and the need for referral. A meeting with the district health team was held to share the project objectives and focus on improving management of sick children in the district. This was followed by meetings within the study clusters with community leaders to obtain their consent to participate.

Subsequently, training of subcounty trainers in the selected clusters was conducted. The specific roles of the trainers were to identify VHTs who would carry out community sensitization to organize training and to supervise VHTs. Two VHT members per village were trained for 6 days using an iCCM module that had the following components: mobilizing and sensitizing mothers and communities; working more closely with the health facilities; emphasizing the importance of having drug storage facilities; looking for sick children; following up children not immunized; recording pregnant mothers; visiting newborns; and drawing up village maps to identify homes with under-fives and reporting under-five deaths.^16^

Village health teams conducted community sensitization before the start of recruitment to inform communities within the study area of the study, its purpose, and what to expect. The VHTs were supervised by the study team, the district health team, and subcounty trainers.

The information relevant to participants during community sensitization activities was summarized in the local language information sheet for VHTs. Simple advertising materials (leaflets) were supplied to private outlets to support community awareness. Both the subcounty trainers and the research team supervised the outlets on a weekly basis in the first 2 months of the interventions, and this was then scaled down over time. No community awareness was conducted in the control arm.

### Public–private partnership building.

Meetings between in-charges of public and private healthcare units in the intervention clusters were convened at public health facilities on a quarterly basis to discuss health system issues focusing on treatment and referral. Discussions were conducted with healthcare providers from the public and private sectors to explore the following themes: treatment and referral practices, community participation, and factors that influenced the treatment and referral of patients. They were held in a meeting room at the public facilities to ensure privacy. Each facility presented one participant except the host facility, where two or three members would be present. Public–private meetings were not convened in the control arm.

### Randomization.

A process of restricted randomization was used to balance cluster-level factors associated with the primary outcome (training in malaria, pneumonia, and diarrhea; and rural/urban location) or those expected to influence the effect of the intervention between the study arms to ensure study credibility and precision.^18^ The STATA^®^ sample command was used to draw the sample.

#### Cluster definition.

A cluster was defined as a parish or neighboring parishes if the distance between any two private outlets located in each of the parishes was < 1 km, to minimize the risk of contamination. A parish is a geographically defined area with a population of at least 5,000 people. In the study district, there were 84 parishes, with 57 deemed eligible from the sampling frame.

#### Balance criteria.

Approximate balance on location (rural/urban) was achieved using proportional allocation: balance to within 10% on the baseline estimate of the primary outcome; balance to within 20% on the three training covariates; proportion with training in the management of malaria; proportion with training in the management of diarrhea; and the proportion with training in the management of pneumonia.

#### Cluster inclusion criteria.

Any of the 84 parishes/clusters in Mukono district were eligible if they 1) contained more than 200 households to ensure a sufficient number of sick children visiting the private outlets, 2) contained at least one drug shop or private clinic registered with the district drug inspector, and 3) contained health center II, the lowest public health facility where early treatment is available. The validity of the restricted randomization was assessed by producing a matrix of the probability that each pair of clusters was allocated to the same study arm.

#### Facility inclusion criteria.

The inclusion criteria were 1) registered drug shops or private clinics and 2) consent to participate in the study.

#### Facility exclusion criterion.

The exclusion criterion was establishment less than 12 months in existence (low number of customers expected).

#### Patient inclusion criteria.

The inclusion criteria were children aged < 5 years with a fever or axillary temperature ≥ 37.5°C, cough, or diarrhea or a history of any of the above when presenting at the private outlet.

#### Patient exclusion criterion.

The patient exclusion criterion was sick children presenting with symptoms other than the aforementioned or being visitors in the village for less than 14 days.

### Study outcomes.

The primary outcome was appropriate case management for malaria, pneumonia, and diarrhea among children in the private sector, whereas the secondary outcome was the proportion of sick children seeking care and receiving prompt treatment at private outlets within 24 hours of onset of symptoms.

Care givers were asked about the presenting signs and symptoms of childhood illnesses, when the illness started, the duration, and treatment seeking elsewhere before coming to the study facilities. Data on patients presenting with a fever or history of fever, cough, and diarrhea were captured prospectively; symptom history, diagnosis, and any treatment received were routinely recorded in a register and study forms provided for this purpose. Data were validated by comparing patient registers, treatment, and referral forms.

### Sample size calculation.

Sample size calculations were performed based on Bennett et al.1991.^19^ A previous study estimated that the proportion of caretakers who completed treatment and referral of a sick child in Uganda was 28%.^20^ Based on this estimate, it was assumed that the intervention would increase the primary outcome from 28% to 40% (size effect of 12%), with a power of 80%, 5% level of significance, standard error of 4%, and a correlation coefficient k of 0.15. With this, a minimum of 10 clusters per arm were calculated. At this power, there were a minimum of 319 sick children per cluster and the total number of sick children targeted for recruitment from all clusters was 8,910, adjusting for a 10% loss to follow-up and possible withdrawal of private outlets.

### Recruitment of patients.

From June 1 2015 to July 31 2017, all private healthcare outlets in both study arms recorded demographic and socioeconomic data on sick children aged < 5 years, type of illnesses, and prescription of drugs. In patient registers, treatment and referral forms were used for data recording.

### Laboratory methods.

An mRDT (First Response^®^, Premier Medical Corp., India) was performed using finger-prick blood. The mRDTs were independently quality-assured by WHO-FIND, Institut Pasteur, Cambodia. Patient record forms and used mRDTs were delivered to the project office in Kampala. An experienced laboratory technician reread used mRDTs and validated the accuracy of mRDT interpretation and reporting by providers.

### Statistical methods.

Data were double entered and verified using Microsoft Access 2007 (Microsoft Inc., Redmond, WA) and analyzed using STATA version 11·0 (STATA Corporation, College Station, TX). Using methods appropriate for a small number of clusters per arm, a modified intention-to-treat cluster level analysis was undertaken.^21^ A measure of the outcome of interest was estimated for each cluster. These cluster-level summaries were analyzed using an unpaired *t*-test to provide risk differences, 95% CIs, and to test the null hypothesis of no intervention effect. Adjustment for covariates was conducted using a two-stage procedure: first, the probability of the outcome of interest was fitted from a regression model including the covariates of interest. Expected values from this model were computed and compared with observed values to provide a difference-residual for each cluster. At the second stage, these cluster-level summaries were analyzed using an unpaired t-test to give adjusted risk differences and 95% CI. Other outcomes of interest were tested using a two-sample proportion test.

## RESULTS

### Background characteristics.

**Figure 1.**
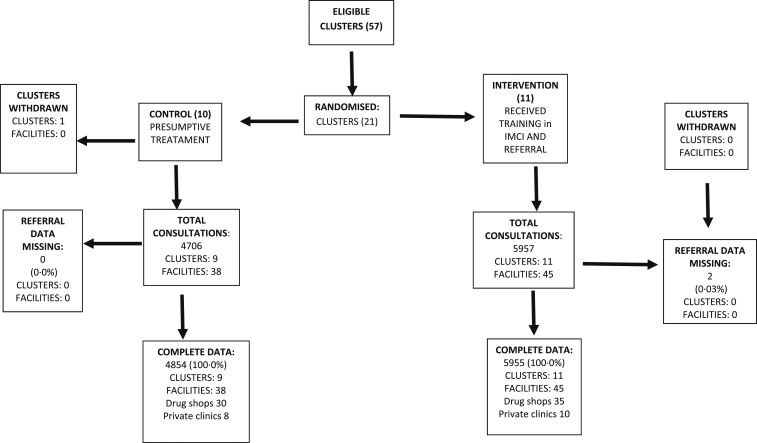
Study trial profile.

The study was implemented in 21 clusters (11 allocated to the intervention and 10 to the control). From these clusters, 83 private health facilities (45 in the intervention arm: 35 drug shops and 10 private clinics; and 38 in the control arm: 30 drug shops and eight private clinics) consented to participate (Figure 1). In the third month of the study, one cluster in the control arm dropped out because of the closure of a drug shop. Consultation data until time of drop out were included in the analysis. A total of 10,809 children aged < 5 years were recruited (5,955 intervention, 4,854 control), with only 20% aged less than 1 year (943 in the control arm and 1,112 in the intervention arm). More than 88% of children had slept under a mosquito net the previous night, and the characteristics of children visiting the two arms were similar (Table 1).

**Table 1:** Characteristics of children who visited private health facilities

	Control arm frequency (%)	Intervention arm frequency (%)
Number of participating parishes (clusters)	9	11
Number of facilities	38	45
Total number of child visits to facilities	4,854	5,955
Age-group (years)*		
< 1.0	943 (19.4)	1,112 (18.7)
1.0–2.9	1,966 (40.5)	2,236 (37.5)
3.0–4.9	1,942 (40.0)	2,599 (43.6)
Gender		
Male	2,374 (48.9)	2,986 (50.1)
Female	2,480 (51.1)	2,969 (49.9)
Slept under a net the previous night		
No	579 (11.9)	614 (10.3)
Yes	4,275 (88.1)	5,341 (89.7)
Resident in the same village as facility		
No	2,862 (59.0)	1,632 (27.4)
Yes	1,988 (41.0)	4,311 (72.4)
Body temperature (°C)	37.6 (37.5–37.6)	37.5 (37.4–37.58)
Weight (kg)	11.8 (11.4–12.2)	13.5 (13.4–13.7)
Day of visit to a facility		
Weekday	3,520 (72.5)	4,396 (73.8)
Weekend	1,334 (27.5)	±1,559 (26.2)

* Data missing for 11 participants (three control, eight intervention).

### Prevalence of childhood illnesses.

The majority of children in the intervention arm 5,003 (84.0%) presented with fever versus 3,791 (78.1%) in the control arm; 3,347 (56.2%) in the intervention arm versus 2,256 (46.5%) in the control arm presented with cough (*P* = 0.0001). Fast breathing was counted in 3,339 (56.1%) children in the intervention arm versus 798 (16.4%) in the control arm (*P* = 0.0001).

In the intervention arm, 2,187 (36.7%) children presented with diarrhea versus 1,232 (25.4%) in the control arm (*P* = 0.0001), and only 1,044 (18%) were dehydrated. In the intervention arm, 2,388 (40.1%) presented to health facilities within the recommended 24 hours, compared with 1,702 (35.1%) who did so in the control arm (*P* = 0.001; Table 2). Malaria rapid diagnostic tests were performed in 5,167 (86.9%) children in the intervention arm (30.1% positive) versus 2,838 (58.5%) in the control arm (60.1% positive) (Table 2).

**Table 2:** Diagnoses and treatment of children who visited private health facilities

	Control arm	Intervention arm	
	Frequency (%)	Frequency (%)	*P*-value
Total number of child visits to facilities	4,854	5,955	
Presenting symptoms			
Fever			
Yes	3,791 (78.1)	5,003 (84.0)	0.0001
Time of visit to facility after onset of symptoms			
Within 24 hours	1,702 (35.1)	2,388 (40.1)	0.001
> 24 hours	3,152 (64.9)	3,567 (59.9)	0.0001
Cough			
Yes	2,256 (46.5)	3,347 (56.2)	0.0001
Diarrhea			
Yes	1,232 (25.4)	2,187 (36.7)	0.0001
Other			
Yes	2,383 (49.1)	2,030 (34.1)	0.0001
Respiratory rate counted			
Yes	798 (16.4)	3,339 (56.1)	0.0001
Breaths per minute	41.3 (40.4–42.2)	43.8 (43.5–44.1)	
Dehydration assessed			
Severe	17 (0.4)	30 (0.5)	0.8
Some	652 (13.4)	1,044 (17.5)	0.0001
Tests performed			
Blood slide taken			
Yes	264 (5.4)	752 (12.6)	0.03
Refused	3 (0.1)	13 (0.2)	–
Results of blood test			
Negative	128 (48.7)	232 (33.8)	0.05
Positive	135 (51.3)	454 (66.2)	
mRDT performed			
Yes	2,838 (58.5)	5,167 (86.9)	0.0001
Refused	362 (7.5)	34 (0.6)	0.13
mRDT result			
Negative	1,124 (39.9)	3,589 (69.9)	0.0001
Positive	1,690 (60.1)	1,548 (30.1)	0.0001
Medication given			
Artemether–lumefantrine			
Yes	1,879 (38.7)	1,973 (33.1)	0.0001
Artesunate			
Yes	31 (0.6)	37 (0.6)	–
Amoxicillin			
Yes	929 (19.1)	2,602 (43.7)	0.0001
Suppository			
Yes	9 (0.2)	8 (0.1)	–
Zinc			
Yes	1,077 (22.2)	2,119 (35.6)	0.0001
Oral rehydration salts			
Yes	1,219 (25.1)	2,112 (35.5)	0.0001
Other treatment*			
Yes	4,300 (88.6)	3,499 (58.8)	0.0001

mRDT = malaria rapid diagnostic test.

* Other treatments included paracetamol, antibiotics, and antimalarial drugs.

### Treatment practices.

Overall, 1,973 (33.1%) children in the intervention arm were given artemether–lumefantrine (Coartem) compared with 1,879 (38.7%) in the control arm, and 31 (0.6%) were given pre-referral rectal artesunate.

Zinc tablets were given to 2,119 (35.6%) of children with diarrhea in the intervention arm compared with 1,077 (22.2%) of children in the control arm, whereas 2,112 (35.5%) in the intervention arm were given ORS compared with 1,219 (25.1%) in the control arm (Tables 2 and 3).

**Table 3:** Treatment of malaria and diarrhea at private health facilities

	AL (%)	Artesunate (%)	No artemisinin-based combination therapy (%)	Total
Control arm				
mRDT result				
Negative	46 (4.0)	4 (0.4)	1,074 (95.6)	1,124
Positive	1,402 (83.0)	17 (1.0)	271 (16.0)	1,690
Intervention arm				
mRDT result				
Negative	432 (12.0)	21 (0.6)	3,136 (87.4)	3,589
Positive	1,459 (94.3)	8 (0.5)	81 (5.2)	1,548
Statistical significance	*P* = 004			

	ORS only (%)	Zinc only (%)	ORS and zinc (%)	No zinc or ORS	Total
ORS treatment by dehydration severity					
Control arm					
Level of dehydration					
No	108 (2.6)	12 (0.3)	578 (13.8)	3,487 (83.3)	4,185
Severe	5 (29.5)	0 (0.0)	9 (52.9)	3 (17.6)	17
Some	61 (9.4)	20 (3.1)	458 (70.2)	113 (17.3)	652
Intervention arm					
Level of dehydration					
No	44 (0.9)	77 (1.6)	1,280 (26.2)	3,480 (26.2)	4,881
Severe	1 (3.3)	0 (0.0)	14 (46.7)	15 (50.0)	30
Some	43 (4.1)	18 (1.7)	730 (69.9)	253 (24.3)	1,044
Statistical significance			*P* = 009		
Treatment of children with diarrhea					
Control arm					
Diarrhea					
Yes	75 (6.1)	21 (1.7)	991 (80.4)	145 (11.8)	1,232
Intervention arm					
Diarrhea					
Yes	24 (1.2)	86 (3.9)	1,948 (89.1)	129 (5.9)	2,187
Statistical significance			*P* = 001		

mRDT = malaria rapid diagnostic test; ORS = oral rehydration salts.

In the intervention arm, 2,602 (43.7%) children received amoxicillin versus 929 (19.1%) in the control arm. Thirty-three (52.4%) children aged 2–11 months with a rapid respiratory rate in the control arm were given amoxicillin, versus 283 (88.4%) in the intervention arm. Similarly, of those aged 1–5 years, 104 (54.5%) children with a rapid respiratory rate in the control arm were given amoxicillin, versus 1,596 (85.1%) in the intervention arm. Two children aged 7 days and 2 months with a normal respiratory rate received amoxicillin in the intervention arm. In children aged 1–5 years, 17 (4.0%) with a normal respiratory rate received amoxicillin in the control arm, versus 241 (27.8%) in the intervention arm (Table 4).

**Table 4:** Treatment of children by respiratory rate at private health facilities

	Amoxicillin given (%)	Total
Control arm		
Rapid respiratory rate by age	137 (53.9)	254
0–7 days, ≥ 60 breaths/minute	0	0
8 days–1.9 months, ≥ 55 breaths/minute	0	0
2–11 months, ≥ 50 breaths/minute	33 (52.4)	63
1–5 years, ≥ 40 breaths/minute	104 (54.5)*	191
Normal respiratory rate by age	25 (4.7)	537
0–7 days, < 60 breaths/minute	0	0
7 days–1.9 months, < 55 breaths/minute	0	0
2–11 months, < 50 breaths/minute	8 (7.2)	111
1–5 years, < 40 breaths/minute	17 (4.0)†	426
Intervention arm		
Rapid respiratory rate by age	1,879 (85.4)	2,199
0–7 days, ≥ 60 breaths/minute	0	0
7 days–1.9 months, ≥ 55 breaths/minute	0	3 (100.0)
2–11 months, ≥ 50 breaths/minute	283 (88.4)	320
1–5 years, ≥ 40 breaths/minute	1,596 (85.1)*	1,876
Normal respiratory rate by age	366 (32.8)	1,115
0–7 days, < 60 breaths/minute	0	0
7 days–1.9 months, < 55 breaths/minute	2 (100.0)	0
2–11 months, < 50 breaths/minute	123 (49.8)	247
1–5 years, < 40 breaths/minute	241 (27.8)†	866

* *P* = 001.

† *P* = 001.

### Adherence to treatment guidelines.

Adherence to mRDT test results was 94.3% (1,459) in the intervention arm compared with 83.0% (1,402) in the control arm (*P* = 0.04; Table 3). Of the children with diarrhea, 1,498 (89.1%) in the intervention arm and 991 (80.4%) in the control arm were given ORS and zinc as recommended in the guidelines (*P* = 0.001; Table 3). Of the children aged 1–5 years with a respiratory rate > 40 breaths/minute, 1,596 (85.1%) in the intervention arm compared with 104 (54.5%) in the control arm were given treatment as the iCCM guideline recommends (*P* = 0.01). In both arms, there was high adherence to the respiratory rate cutoff points and to diarrhea treatment guidelines, with no statistical differences between the arms (Table 4). Appropriate treatment of mRDT-positive children with artemether–lumefantrine (Coartem) was administered to 93.1% (95% CI 88.5–97.7) in the intervention arm compared with 85.1% (95% CI 78.6–91.7) in the control arm (*P* = 0.03). Seeking care within 24 hours of onset of symptoms occurred with 45.4% (95% CI 36.0–48.8) of children in the intervention arm compared with 43.9% (95% CI 38.1–49.8) in the control arm (*P* = 0.04; Table 5).

**Table 5:** Treatment of sick children in private health facilities

	Control arm frequency (%)	Intervention arm frequency (%)				
Number of participating parishes (clusters)	9	11				
Number of facilities	38	45				
Total number of child visits to facilities	4,854	5,955				
Total number of children with referral data	4,854	5,955				
Total number of children with mRDT adherence data	2,814	5,137				
Total number of children with respiratory rate data	791	3,314				
Total number of children with diarrhea data	4,744	5,882				
Total number of children with treatment seeking data within 24 hours	4,128	5,262				

Trial endpoints	Cluster mean (95% CI)	Cluster mean (95% CI)	Unadjusted risk difference (95% CI)	*P*-value	Adjusted risk difference (95% CI)	*P*-value
Appropriate treatment (giving artemisinin-based combination therapy to mRDT-positive children)	85.1 (78.6–91.7)	93.1 (88.5–97.7)	8.0 (7.0–15.2)	0.03	7.7 (5.0–14.9)	0.03
Seeking care at private outlets within 24 hours of onset of symptoms	43.9 (38.1–49.8)	45.4 (36.0–48.8)	1.5 (1.2–6.7)	0.04	1.9 (1.6–6.3)	0.04

mRDT = malaria rapid diagnostic test.

## DISCUSSION

The present study evaluated an intervention to improve treatment practices for sick children in the private health sector. Our findings show that a high number of sick children were enrolled in the study and trained private providers adhered to treatment iCCM guidelines.

The study had three components: training providers to treat childhood illnesses, raising community awareness on the importance of prompt care seeking, and linking up private and public providers in a dialogue to discuss health system issues. We believe these components were implemented successfully because training private healthcare providers was accompanied by a high proportion of them treating malaria, diarrhea, and pneumonia appropriately through adhering to the guidelines. For example, fast breathing among sick children was counted by a higher proportion of providers in the intervention arm compared with the control arm; this may be attributed to training the providers and equipping them with respiratory timers.

Prompt care seeking was, however, moderate in both arms, indicating that awareness on the importance of seeking care within 24 hours of onset of symptoms and decision-making at a household level were probably constraints as previously reported.^11^

The results are generalizable to most parts of Uganda where malaria, pneumonia, and diarrhea are prevalent among children aged less than 5 years, and the first resort to treatment seeking is at the private health facilities (drug shops and private clinics). In addition, the patient population included all sick children seeking treatment for fever, diarrhea, and pneumonia, and, therefore, is representative of the population of children who seek care at private health facilities. Because this was a randomized study, it is reasonable to assume that health risks of patients were similar. It should be noted that most districts in Uganda have the same rural–urban distribution of private health facilities, as well as registration status. We, therefore, think that these results could be generalized to many areas in Uganda with the same endemicity of childhood illnesses.

These results are consistent with previous findings in the district that revealed that introducing mRDTs in drug shops improved appropriate treatment of malaria, reduced overprescription of antimalarial drugs and adherence to mRDT results.^8^ It has also been shown that training and supervising drug shops improved treatment of childhood illnesses and increased access to essential care.^22–24^ In another study to improve antimalarial prescription in drug shops in Uganda, it was shown that perceived increase in professional status, patient numbers, and profitability; supervisory visits by the Ministry of Health; and reduced fear of closure of shops were influential factors among drug shop vendors and helped to promote adherence to prescribing guidelines.^25^

In Tanzania, it has been shown that accredited and supervised drug shops increased access to drugs and encouraged more pediatric formulations compared with health facilities.^26^ Similarly, training and supervising drug shops in rural Tanzania showed that they performed mRDTs to two-thirds of suspected malaria patients, and the study concluded that introducing mRDTs into regulated private retail sector settings improved malaria testing and treatment practices.^27^ In Ghana, providing mRDTs in the private drug retail sector significantly reduced dispensing of antimalarial drugs to patients without malaria.^28^

An important finding in the present study is that a high proportion of children with a normal respiratory rate were given amoxicillin. This is probably attributed to iCCM training where children were assessed and given treatment based on clinical symptoms of fever, cough, respiratory rate, and a negative mRDT test that encouraged providers to give an antibiotic. It is possible that providers may have felt compelled to provide some form of treatment to a sick child whose mRDT was negative. This may have led to a greater likelihood of unnecessary antibiotic prescription. In addition, there may have been a profit motive behind this practice. It is also possible that the skills of providers deteriorated over time, affecting quality of care. However, this observation is consistent with recent findings in the study area and elsewhere that revealed that introducing mRDTs to diagnose and treat malaria is associated with increased use of antibiotics.^29^ Also, it should be noted that in the control arm, a large number of mRDTs were performed. This is because before the implementation of the study, the Ministry of Health rolled out mRDTs at the community level including the private sector in the district. This roll-out was accompanied by awareness-raising on the importance of adherence to the mRDT test.^9^

We also note that the mRDT positivity rate in intervention arm was 30% compared with 60% in the control arm. Because the intervention was randomized and provider background characteristics like prior training and experience evenly distributed, the observed difference could be attributed to the iCCM training in intervention arm. Providers in the intervention arm were trained and supported to master skills for 2 months, given SOPs to adhere to intervention procedures, and were more likely to perform clinical assessment of children, perform mRDTs and interpret the results better, and provide treatment according to guidelines. However, there is need to discuss these results with Ministry of Health program managers and development partners involved in scaling up iCCM to ensure quality assurance by intensifying monitoring and support supervision.

Prompt care seeking within 24 hours was measured by taking history of onset of symptoms and the time of reporting to the study facilities. No measure of delays at home, reaching the facilities, and at the facilities was carried out. This could have provided insights into constraints to accessing timely care. We recommend this for future studies. Frequent stock-outs of drugs and supplies, poor customer care, and absenteeism of staff at study facilities had been reported in the baseline study.^1^ However, addressing these constraints was beyond the capacity of this study; future interventions should address this important aspect for improving treatment and referral uptake.

The policy implications of these findings are to initiate a dialogue at district and national levels on how to scale up the intervention in the private sector. Because Uganda has a public–private partnership policy that supports this initiative, resources should be identified to further support this intervention. Public–private dialogues should be sustained to discuss and identify solutions to health system constraints, especially for improving quality of care in both sectors.

In conclusion, training private healthcare providers improved treatment of malaria, pneumonia, and diarrhea because of provider adherence to treatment guidelines. Further studies are needed to identify opportunities and barriers to scale up this intervention.
